# Activation of Blood Vessel Development in Endometrial Stromal Cells In Vitro Cocultured with Human Peri-Implantation Embryos Revealed by Single-Cell RNA-Seq

**DOI:** 10.3390/life11050367

**Published:** 2021-04-21

**Authors:** Bo Lv, Xiaoyu Xu, Xunyi Zhang, Lingbin Qi, Wen He, Lu Wang, Xian Chen, Luying Peng, Jinfeng Xue, Yazhong Ji, Zhigang Xue

**Affiliations:** Tongji Hospital, School of Medicine, Tongji University, Shanghai 200092, China; lvbo@tongji.edu.cn (B.L.); 1831127@tongji.edu.cn (X.X.); zxymeng@aliyun.com (X.Z.); qlbin@tongji.edu.cn (L.Q.); 1811287@tongji.edu.cn (W.H.); 1831126@tongji.edu.cn (L.W.); 1810444@tongji.edu.cn (X.C.); luyingpeng@tongji.edu.cn (L.P.)

**Keywords:** endometrial stromal cells, cocultured embryos, implantation, blood vessel development, stromal–trophoblast interaction

## Abstract

In humans, the maternal endometrium participates in the physical and physiological interaction with the blastocyst to begin implantation. A bidirectional crosstalk is critical for normal implantation and then a successful pregnancy. While several studies have used animal models or cell lines to study this step, little knowledge was acquired to address the role of endometrial cells in humans. Here, we analyzed single-cell sequencing data from a previous study including 24 non-coculture endometrial stromal cells (EmSCs) and 57 EmSCs after coculture with embryos. We further explored the transcriptomic changes in EmSCs and their interactions with trophoblast cells after coculture. Differentially expressed gene (DEG) analysis showed 1783 upregulated genes and 569 downregulated genes in the cocultured embryos. Weight gene coexpression network and gene ontology analysis of these DEGs showed a higher expression of *RAMP1*, *LTBP1*, and *LRP1* in EmSCs after coculture, indicating the enrichment of biological processes in blood vessel development and female pregnancy. These data imply that EmSCs start blood vessel development at the implantation stage. Compared with endometrium data in vivo at the implantation window, key pathways including epithelial cell development and oxygen response were involved at this stage. Further analysis using CellphoneDB shed light on the interactions between EmSCs and embryonic trophoblasts, suggesting the important role of integrins and fibroblast growth factor pathways during implantation. Taken together, our work reveals the synchronization signaling and pathways happening at the implantation stage involving the acquisition of receptivity in EmSCs and the interaction between EmSCs and trophoblast cells.

## 1. Introduction

Implantation is a key event for embryos to continue development [1]. Successful implantation relies on the synchronized development of the endometrium and embryo [2]. Human embryos penetrate and begin to invade into endometrial cells during the “window of implantation” (WOI) [3]. This process requires delicate spatiotemporal coordination by feto-maternal crosstalk, whose failure is considered as the major reason for implantation failure [4]. Furthermore, formation of blood vessels or angiogenesis in the endometrium is generally considered important for nutrient and oxygen transport between the mother and fetus [5]. Notably, stromal cells undergoing decidualization, which interact with epithelial cells, maternal blood vessels, and embryonic trophoblast cells, play crucial roles in recruiting development factors and supporting embryo implantation. However, the implantation rate is still lower than expected in natural and assisted reproduction events, and little is known about this process in humans.

Considering the unethical and impractical reality of manipulating the human embryo in vivo, approaches to study implantation involve either inference from animal embryo implantation data or the culture of human peri-implantation embryos in vitro [6]. Recently, two studies developed a new in vitro culture system for human post-implantation embryos [7,8]. Moreover, the in vitro coculture of human embryos with endometrial cells offers us a better way of understanding their interaction [6,9]. Our group and Zhou et al. [10,11] recently published studies focusing on the development of the trophectoderm in peri-implantation conceptuses using coculture and single-cell RNA sequencing methods, respectively.

In the present study, we compared EmSCs and trophectoderm cells before and after coculture using the coculture model in vitro, whereby the implantation progress mimicked that in vivo. By analyzing the gene expression profiles, we found significant changes in the EmSCs after coculturing with human embryos compared to the non-cocultured EmSCs. Subsequently, weighted gene coexpression network analysis (WGCNA) and gene ontology (GO) analysis were also applied to analyze the differentially expressed genes (DEGs). The underlying interactions between embryos and EmSCs at these stages were also explored to gain insight into the implantation process and identify potential biomarkers for diagnosis, prognosis, and drug targeting for pregnancy-related malfunctions.

## 2. Materials and Methods

### 2.1. Coculture Model and Sample Collection

Human embryos and endometrium tissue were donated by couples with no genetic disorders or diseases from standard in vitro fertilization (IVF) treatment at the Reproductive Medicine Center, Tongji Hospital, with written informed consent. The primary endometrium was dissected using scalpels and then enzymatically digested in 1% collagenase I and IV (Gibco, 17100-017 and 17014-019) by repeatedly pipetting up and down for 30 min at 37 °C. After filtration through 40 μm strainers and centrifugation, the pellet at the bottom was washed twice with phosphate-buffered saline (PBS). Then, the pellet was resuspended, plated, and prepared for use.

The procedure of culturing embryos was modified and performed according to previously published studies [7,8]. Briefly, pre-implantation human embryos were cultured according to a previous procedure [12]. After embryos hatched from the zona pellucida (late blastocyst stage, day 6.5), we transferred them to the dishes with plated endometrial cells and then cocultured them for 2.5 days. The embryos and endometrial cells underwent incubation with 0.25% trypsin before single cells were dissociated with repeated aspiration through a mouth-operated, drawn capillary pipette. Individual endometrial cells and embryonic cells were placed into a 200 μL PCR tube, and single-cell RNA sequencing (RNA-Seq) was performed using the Smart-seq2 method [13].

### 2.2. Transcriptome Data Processing and Validation

Single-cell RNA-Seq data were processed as follows: TrimGalore! was used to trim the fastq data [14]. Then, the trimmed data were aligned to human reference genome hg38 using STAR (v2.6.0) with default parameters [15], followed by read counting using featureCounts v1.6.2 [16]. The cocultured embryonic cells and endometrial cells were separated using unbiased clustering, as described in our previous work [10]. The trophoblast cells were characterized using 300 epiblast, primitive endoderm, and trophectoderm markers [17] identified in previous work [10]. The endometrial cells, including those before and after coculturing with embryos, were identified as EmSCs using the stromal marker, vimentin (VIM) (Figure S1, Supplementary Materials). The EmSCs before coculturing were defined as “PRE” and the EmSCs after coculturing were defined as “POST”. Principal component analysis (PCA), unsupervised hierarchal clustering, and DEG identification were carried out using the DEseq2 package (v1.24.0) [18]. Transcript per million (TPM) was used to normalize the raw read count for subsequent WGCNA analysis [19]. The transcriptomes of endometrium tissue at WOI and before WOI were downloaded from Hu et al. [20]. The trophoblast cells in the cocultured embryo and the cocultured EmSCs were used for CellPhone DB analysis [21]. Genes with a *p*-value < 0.05 and fold change ≥2 were characterized as DEGs. TB GreenTM Premix Ex TaqTM II (Takara, RR820A) and a Roche Light Cycler 96 system (Roche) were used to carry out qRT-PCR. Relative levels of RNAs were calculated using the ddCt method with *GAPDH* as an endogenous control. The primers are listed in Table S3 (Supplementary Materials).

### 2.3. Weighted Gene Coexpression Analysis (WGCNA)

WGCNA was performed on normalized gene expression data measured using the TPM metric, with 2352 DEGs determined by the DEseq2 package. WGCNA was performed according to a previously published protocol [19]. Briefly, a topological overlap matrix (TOM) was constructed with softPower set to three. The relationships between different modules were visualized using the module and eigengene relation heatmap. Genes contained in the modules were extracted and analyzed using Metascape for a precise representation of the data [22]. The gene coexpression network was extracted and further processed using MCODE in Cytoscape software (V3.7.2) [23].

### 2.4. Gene Ontology (GO) and Gene Set Enrichment Analysis (GSEA)

GO enrichment analysis was performed on a platform combining the R package “clusterProfiler” [24] and the Database for Annotation, Visualization, and Integrated Discovery (DAVID) online tool (https://david.ncifcrf.gov/ (accessed on 13 January 2021)) [25]. Terms with a *p*-value < 0.05 were counted as statistically significant. GSEA was performed to detect the key gene sets under the condition of embryo coculture in the datasets of whole EmSCs [26]. Boxplots were utilized to illustrate selected key genes using the ggplot2 package (V3.2.1) [27]. The TPM values of selected genes were evaluated using Mann–Whitney *U* tests.

## 3. Results

### 3.1. Identification of DEGs and Enriched Pathways after Implantation

**Figure 1:**
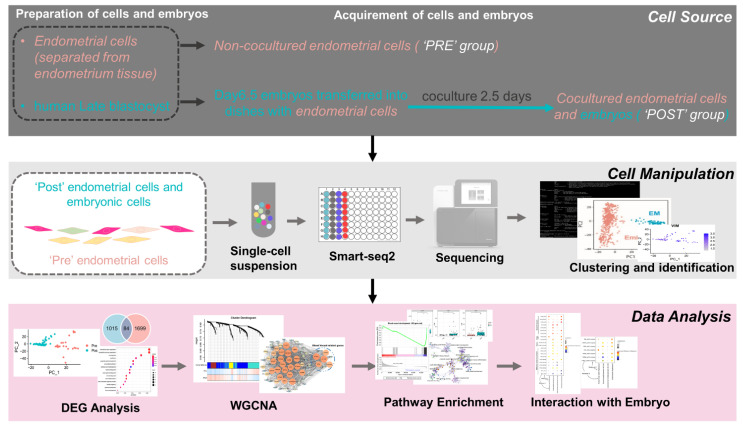
Overview of our study from the preparation and acquisition of embryonic and endometrial stromal single cells to the manipulation and analyses of transcriptomic data. Cell source: after embryos hatched from the zona pellucida (embryonic day 6.5), they were transferred to dishes with plated endometrial cells and then cocultured for 2.5 days. Single cells from embryos and endometrial cells were dissociated with trypsin. Cell manipulation: cocultured and non-cocultured endometrial cells were subjected to the Smart-seq2 method. Cocultured endometrial cells were first separated from embryonic cells using the unbiased clustering method described in our previous report [10] and then characterized as endometrial stromal cells (EmSCs) using the stromal cell marker. Data analysis: differentially expressed genes (DEGs) between “POST” (after coculturing) and “PRE” (before coculturing) EmSCs were analyzed using DEseq2 and further subjected to weighted gene coexpression network analysis (WGCNA), gene set enrichment analysis (GSEA), and gene ontology (GO) analysis.

To explore the potential factors involved in the interactions between blastocysts and EmSCs, the non-coculture EmSCs and EmSCs cocultured with day 6.5 pre-implantation blastocysts were included in this study (Figure 1). Using the smart-seq2 method, we collected 24 “PRE” EmSCs and 57 “POST” EmSCs for single-cell RNA-Seq (Figure S1 and Table S1, Supplementary Materials). 

**Figure 2:**
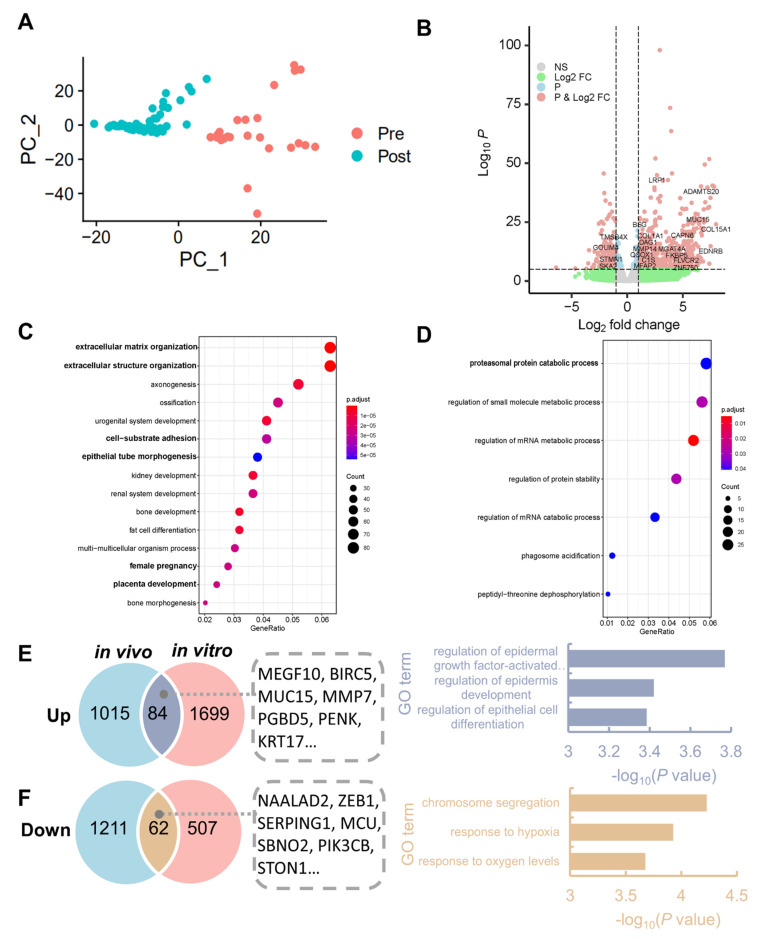
Identification of DEGs and enriched pathways in EmSCs before and after coculture with embryos. (**A**) Principal component analysis (PCA) and clustering of EmSCs. (**B**) Volcano plot of DEGs between the “PRE” group and the “POST” group of EmSCs; “NS” indicates genes with no significant difference *(p* ≥ 0.05) and abs(fold change) <2 between two groups; “Log2 FC” indicates genes with no significant difference (*p* ≥ 0.05) and abs(fold change) ≥2 between two groups; “P” indicates genes with a significant difference (*p* < 0.05) and abs(fold change) <2 between two groups; “P and Log2 FC” indicates genes with a significant difference *(p* < 0.05) and abs(fold change) ≥2 between two groups. (**C**) GO analysis of upregulated genes in the “POST” EmSCs. (**D**) GO analysis of downregulated genes in the “POST” EmSCs. (**E**,**F**) Venn diagram and GO analysis of upregulated genes (**E**) and downregulated genes (**F**) in in vivo endometrial tissue (at window of implantation (WOI) vs. before WOI) and in vitro EmSCs.

PCA and unsupervised hierarchal clustering analyses revealed that EmSCs in the “PRE” group and the “POST” group formed distinct clusters (Figure 2A). Furthermore, among the total of 2352 DEGs in EmSCs found between the “PRE” group and “POST” group using DEG analysis (false discovery rate (FDR) <0.05 and fold change ≥2), 1783 genes, e.g., *LRP1*, *MUC15*, and *COL15A1*, were upregulated and 569 genes, e.g., *TMSB4X*, *STMN1*, and *GOLIM4*, were downregulated in the “POST” group (Figure 2B and Table S1, Supplementary Materials), followed by downstream single-cell transcriptomic analysis (Figure 1).

To further elucidate the mechanism underlying changes in EmSCs during the implantation process, GO analysis of the DEGs was performed to investigate the overrepresented signals. The results showed that the 1783 upregulated genes in the “POST” group were significantly enriched in biological processes such as extracellular matrix organization, cell–substrate adhesion, female pregnancy, and placenta development (Figure 2C). Furthermore, the 569 downregulated genes were also subjected to GO analysis. Intriguingly, most GO items were related to RNA catabolic pathways (Figure 2D), indicating the reservation of RNA in the EmSCs to further embryo embedding. Taken together, these results suggest the activation of extracellular matrix pathways in EmSCs to support embryo implantation.

Since a previous study compared the transcriptome of whole endometrium tissue at the receptive stage to the pre-receptive stage in the normal menstruation cycle [20], we sought to determine whether there were any common changes in endometrial cells between in vivo results during WOI without embryo invasion and our in vitro results with embryo invasion. The DEGs at the receptive stage [20] and the DEGs after implantation in our study [10] were analyzed. Interestingly, we found 84 shared upregulated genes and 62 shared downregulated genes (Figure 2E,F). The 84 shared upregulated genes, including *MMP7*, *BIRC5*, *MUC15*, and *MEGF10*, were then subjected to GO analysis to explore the pathways involved during implantation. These upregulated genes were preferentially enriched in the regulation of epidermal growth factor-activated receptor activity, epithelial cell differentiation, and epidermis development (Figure 2E), while the 62 downregulated genes were enriched in GO terms such as chromosome and hypoxia pathways (Figure 2F). It was reported that *BIRC5* acts as a target of interleukin 11 (IL11) during decidualization [28], whereas *MUC15* and *MMP7* mediate the invasion of the trophoblast into the endometrium [29,30], and MEGF10 plays an adhesion role according to an in vitro experiment [31]. Overall, these results indicate the several distinct pathways activated only in EmSCs with the event of embryo implantation.

### 3.2. Genetic Programs of Receptivity Development in EmSCs Revealed by WGCNA

**Figure 3:**
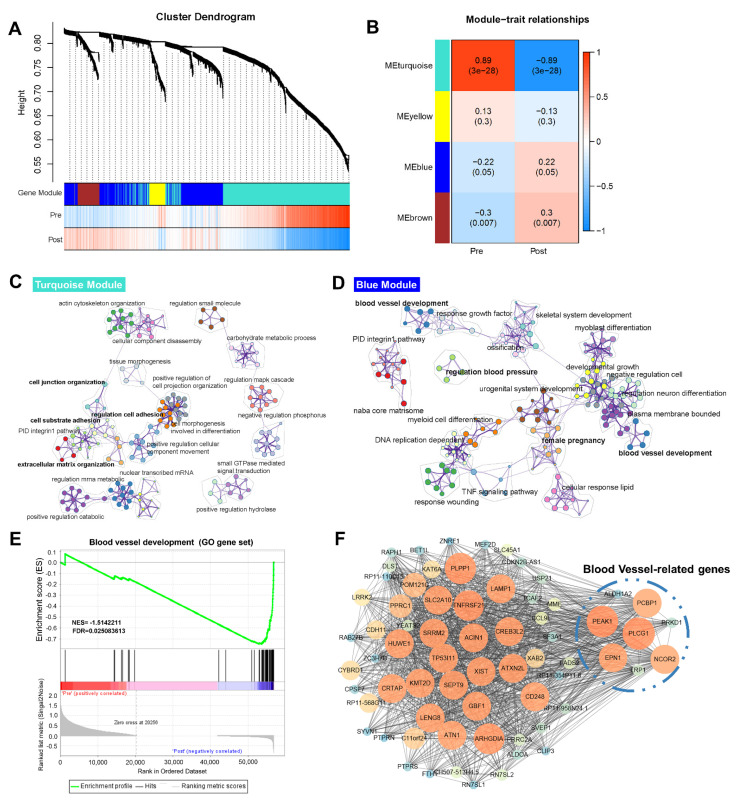
Genetic programs of receptivity development in the EmSCs revealed by WGCNA. (**A**) The dendrogram of gene modules built by WGCNA. Bars represent the correlation between genes and gene modules. Red lines denote a positive correlation and upregulation; blue lines denote a negative correlation and downregulation. (**B**) Module–trait relationships among the four modules. The number in each cell represents the degree of correlation. Different colors represent different gene modules. (**C**,**D**) Network of enriched GO terms in genes contained in the turquoise module (**C**) and blue module (**D**). (**E**) GESA for the gene set of blood vessel development. The enrichment score (ES) describes the value of maximum deviation from 0 for the running sums. Each line in the middle plot represents the different genes. The ranked list metric describes the values of correlation to the cells. (**F**) Cytoscape illustration of the coexpression network of genes in the blue module. The size and the orange color of nodes positively represent the MCODE score of genes.

PCA showed that EmSCs were grouped by “PRE” and “POST” states (Figure 2A). To systematically investigate the genetic program dynamics, we performed WGCNA on the 2352 DEGs in EmSCs between the “PRE” group and “POST” group. WGCNA identified four gene modules, each of which contained a set of genes that tended to be coexpressed with the stage (Figure 3A). By studying module–trait relationships, we found that two gene modules, i.e., the turquoise module and the yellow module, were positively correlated with the “PRE” state, while the other two, i.e., the blue module and the brown module, positively correlated with the “POST” state (Figure 3B). These modules represent the core genetic programs that operate during EmSC development at the embryo implantation stage. We then performed GO analysis on the genes in each module to investigate their biological function (Figure 3C,D). In the turquoise module, genes were mainly enriched in cell junction organization, extracellular matrix organization, and other functions (Figure 3C), while blood vessel development, female pregnancy, and regulation of blood pressure were abundant in the blue module (Figure 3D).

**Figure 4:**
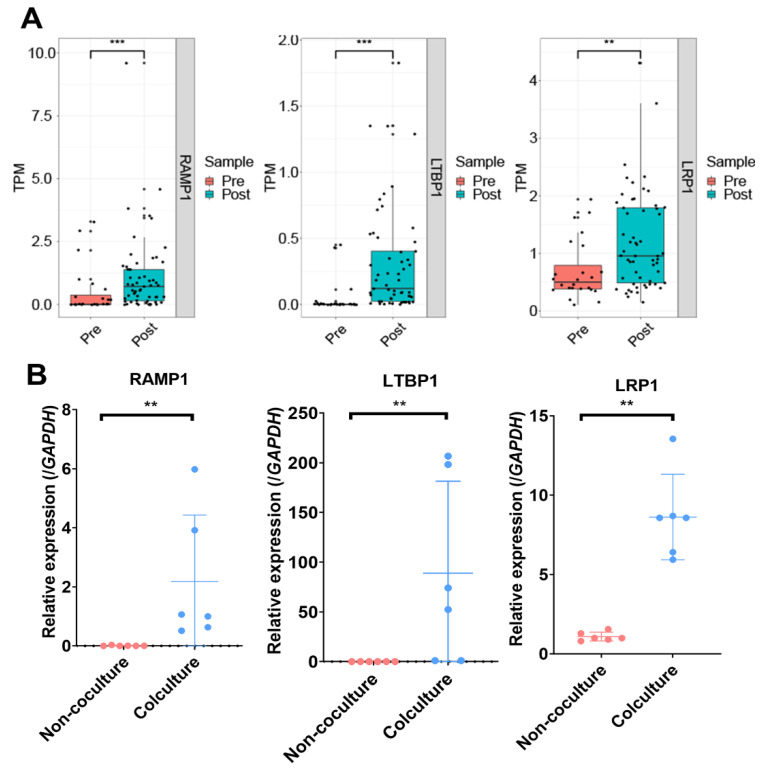
Three DEGs (*RAMP1*, *LTBP1*, and *LRP1*) were related to blood vessel development between the “PRE” EmSCs and the “POST” EmSCs. (**A**) Single-cell RNA data shown in a boxplot using transcript per million (TPM). (**B**) qPCR validation of the three genes in cocultured and non-cocultured EmSCs (*n* = 6). The asterisks denote statistical significance (** *p* < 0.01, *** *p* < 0.001). Dots represent the values of individual cells in each group.

To further validate whether the pathways described above were significantly activated after coculturing with embryos, GSEA was applied, whereby it was found that the item “blood vessel development” from the GO gene set was significantly enriched in the “POST” group (Figure 3E; Table S2, Supplementary Materials), suggesting a significant difference in blood vessel development after coculturing. Moreover, the only genes in the blue module positively related to the EmSCs of the “POST” group were enriched in blood vessel development (Figure 3D). Therefore, we further analyzed the genes in the blue module using the Cytoscape MCODE plugin. The hub genes with a high MCODE score were related to many functions during cell growth and receptivity establishment, including histone modification (*KAT6A* and *KMT2D*) [32,33], inhibition of endometriosis development (*TNFRSF1A*) [34], inhibition of inflammatory response (*POM121C*) [35], and regulation of epithelial–mesenchymal transition (*CDH11*) [36]. Intriguingly, a subset of genes related to blood vessel development was identified, including *EPN1*, *PEAK1*, *PCBP1*, *PLCG1*, and *NCOR2* (Figure 3F), which play important roles in hematopoietic stem-cell emergence, endothelial cell recruitment, and vascular branching [37,38,39,40,41]. Revealing the dynamics of blood vessel-related genes impacted by embryos, genes such as *RAMP1*, *LTBP1*, and *LRP1* showed significantly higher expression in the “POST” group than in the “PRE” group (Figure 4A). This was then verified by quantitative reverse-transcription polymerase chain reaction (Figure 4B). Overall, these findings suggest that blood vessel development might be activated in endometrial cells at the stage of embryo implantation.

### 3.3. Crosstalk between Endometrium and Trophoblast Cells during Implantation

**Figure 5:**
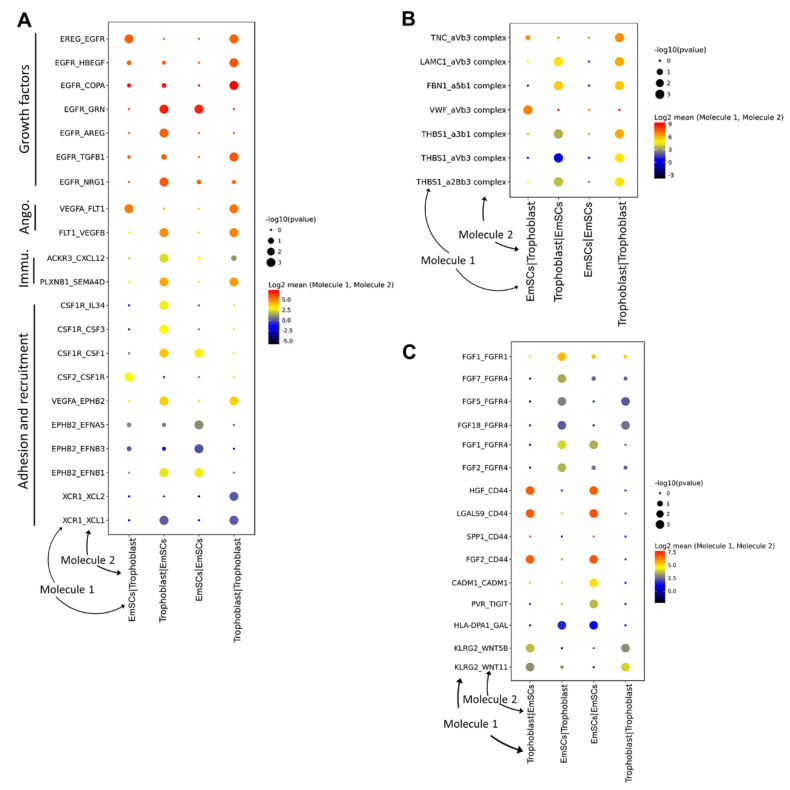
Ligand–receptor interaction analysis of the EmSCs and trophoblast cells after coculture using CellphoneDB. (**A**) Interactions reported by Vento-Tormo et al. (**B**,**C**) integrins (**B**) and fibroblast growth factors (FGFs), cluster of differentiation 44 (CD44), FGF receptors, and other potential interactions (**C**) involved in EmSC–trophoblast cell–cell interactions.

To systematically analyze the interactions between EmSCs and trophoblast cells, we used CellphoneDB [21] to identify the expression of possible crosstalk. These interactions were calculated at the transcriptomic level but extrapolated to protein activity. Similar to a previous report using the first-trimester placenta and decidua [21], we found that growth factors, angiogenesis proteins, and adhesion and recruitment ligands and receptors were highly expressed in the EmSCs and the trophoblast cells during implantation, indicating that these events existed from implantation to the early pregnant placenta (Figure 5A).

Furthermore, integrins are reported as one of the most important proteins regulating blastocyst implantation [42]; thus, we sought to determine which integrin and its target were expressed in these two cell types. Interestingly, the EmSCs at the implantation stage highly expressed *TNC* and *VWF*, while their receptor integrin *aVb3* was highly expressed in the trophoblast cells with relatively low expression in the EmSCs themselves (Figure 5B). On the other hand, *LAMC1*, *FBN1*, and *THBS1* were expressed at a high level in the trophoblast cells, while their receptors (*aVb3*, *a5b1*, and *a3b1*) were also highly expressed in EmSCs (Figure 5B). In addition to integrins, we found a high expression of fibroblast growth factors (FGFs) and cluster of differentiation 44 (CD44) in EmSCs together with a high expression of FGF receptors and CD44 ligands (*HGF*, *LGALS9*, and *FGF2*) in trophoblast cells (Figure 5C), further indicating the preparation of blood vessel establishment. Taken together, these patterns of ligand–receptor expression strongly suggest complex interactions between the EmSCs and trophoblast cells, providing additional insight into the implantation process.

## 4. Discussion

In this study, we explored the influence of human embryos on EmSCs by characterizing the DEGs after implantation at the single-cell level. Despite no decidualization-induced treatment being applied to the EmSCs before coculturing in this study, the EmSCs after coculture showed an activated extracellular matrix and cell–substrate adhesion, highlighting the similarity in stromal cell activity during decidualization at the WOI [43] (Figure 2E,F). Of note, Wang et al. published their work using endometrial single cells during the whole menstrual cycle [44]. We also compared our single-cell data with theirs. Intriguingly, proliferative-phase genes, such as *STC1*, *NFATC2*, *BMP2*, and *PMAIP1*, were downregulated in the “POST” EmSCs compared to the “PRE” EmSCs (Figure S4, upper panel, Supplementary Materials), while they were also downregulated at the secretory phase [44]. However, secretory-phase genes, such as *LMCD1*, *FGF7*, and *IL15*, showed no difference in the cocultured EmSCs as compared with non-cocultured cells, whereas another secretory-phase gene, *CRYAB*, even showed higher expression before coculture (Figure S4, lower panel, Supplementary Materials). These in vitro results suggest changes in stromal cells from the proliferative phase to the secretory phase; however, these changes are not completely possible due to the different states of stromal cells cultured in vitro and the relatively short coculture time with embryos.

Implantation is one of the most important processes for successful pregnancy [1]. Synchronization between the acquisition of invasion competency in embryos and receptivity ability in the endometrium is required for implantation. Consistent with previous findings [21], our study indicates that the endometrium prepared a receptive state for embryo implantation, as upregulated genes in the “POST” group were enriched in biological processes such as cell junction and adhesion, extracellular matrix organization, and female pregnancy (Figure 2C,D). WGCNA is a useful tool to detect specific modules [19]. By combining this tool with GO, blood vessel development was identified from the blue module (Figure S2A and Table S2, Supplementary Materials). The hub genes in this module were also found to be positively related to the “POST” EmSCs (Figure 3F and Figure 4). Both vasculogenesis and angiogenesis are of crucial importance during blood vessel development when the embryo implants [5]; accordingly, we found that many genes related to hematopoietic stem-cell emergence, cell growth factors, and chemokines, which were highly expressed in the “POST” EmSCs, were all highly correlated with blood vessel establishment (Figure 3 and Figure 4; Figures S2B and S3 and Table S2, Supplementary Materials). These results are consistent with the function of the endometrium at the peri-implantation stage, when the uterine spiral arteries begin remodeling to achieve circulation characteristic for successful placentation and the fetal–maternal interface [1,3].

The two-way interactions between the endometrium and trophoblast initiate the implantation process after the embryo enters the uterus [45]. CellphoneDB (created by Vento-Tormo et al.) has shown its power in analyzing cell–cell interactions [21]. The number of immunomodulation pathways in our study, including the atypical chemokine receptor 3 (ACKR3)/C–X–C motif chemokine ligand 12 (CXCL12) pathway, was lower compared to the first-trimester placenta data, including programmed death ligand 1 (PDL1)/programmed death 1 (PD1) and adenosine A2B receptor (ADORA2B)/CD39 [21], which was probably due to the limited cell types included in our studies. However, other ligands/receptors that might regulate the implantation event were found to be highly expressed, such as the T-cell immunoreceptor with immunoglobulin (Ig) and immunoreceptor tyrosine-based inhibitory motif (ITIM) domains (TIGIT)/poliovirus receptor (PVR) pathway, which can inhibit human immune cell activity and might benefit implantation [46]. Moreover, killer cell lectin-like receptor G2 (KLRG2), responsible for regulating decidualization and embryonic development, possibly through the Wnt pathway [47], was also highly expressed in our study (Figure 5C). Overall, our study provides insight into the endometrial stomal cell state transition during embryo implantation in vitro, showing that receptivity is established after embryo coculture. These findings can help us better understand the intercellular events during implantation.

However, our study also had some limitations. Since it is unpractical and unethical to acquire human embryos in vivo during implantation, our study mimicked the implantation process by applying EmSCs to the post-implantation culture method developed by Deglincerti et al., and Shahbazi et al. [7,8]. Therefore, the actual implantation process, with a more complex environment and more cell types in vivo, may undergo different cellular pathways, several of which were shown in our study (Figure 2E,F; Figure S4, Supplementary Materials). Moreover, the differential expression of blood vessel-related genes presented in our study was analyzed at the transcriptomic level, and further validations such as quantitative real-time PCR and/or immunostaining of key pathway signals are required to understand the activity of receptivity state transition.

In conclusion, our work provides one piece of the jigsaw puzzle for a better understanding of human embryo implantation, using the in vitro coculturing model of human embryos and EmSCs, which highlighted not only synchronization in signaling pathways and regulatory networks happening in the EmSCs but also an interaction between peri-implantation trophoblast cells and EmSCs.

## Data Availability

The transcriptome data are available from the Gene Expression Omnibus (GEO) database with accession number GSE125616.

## Supplementary Materials

The following are available online at https://www.mdpi.com/article/10.3390/life11050367/s1: Table S1. Summary of number of individual EmSCs and DEGs between PRE and POST group; Table S2. The list of gene sets used in Figure 3E (“blood vessel development”) and Figure S2 (“female pregnancy” and “angiogenesis”); Table S3. The list of primers used in the study; Figure S1. Characterization of endometrial stromal cells. Plot of vimentin (VIM) expression among all cells (A) and between the ‘PRE’ EmSCs and the ‘POST’ EmSCs (B); Figure S2. GSEA for female pregnancy gene set (A) and angiogenesis gene set (B).

## Abbreviations

EmSCs: endometrial stromal cells; WGCNA, weighted gene coexpression network analysis; GO, gene ontology; DEG, differentially expressed genes; PCA, principal component analysis; WOI, window of implantation; TPM, transcript per million; TOM, topological overlap matrix; GSEA, gene set enrichment analysis; FDR, false discovery rate; VIM, vimentin; MMP7, matrix metallopeptidase 7; BIRC5, baculoviral IAP repeat-containing 5; MUC15, mucin 15; COL15A1, collagen type XV alpha 1 chain; TMSB4X, thymosin beta 4 X-linked; STMN1, Stathmin 1; GOLIM4, Golgi integral membrane protein 4; MEGF10, multiple EGF-like domains 10; KAT6A, lysine acetyltransferase 6A; KMT2D, lysine methyltransferase 2D; TNFRSF1A, TNF receptor superfamily member 1A; POM121C, POM121 transmembrane nucleoporin C; CDH11, cadherin 11; EPN1, Epsin 1; PEAK1, pseudopodium enriched atypical kinase 1; PCBP1, poly(RC)-binding protein 1; PLCG1, phospholipase C gamma 1; NCOR2, nuclear receptor corepressor 2; RAMP1, receptor activity-modifying protein 1; LTBP1, latent transforming growth factor beta-binding protein 1; LRP1, LDL receptor-related protein 1; TNC, tenascin C; VWF, Von Willebrand factor; LAMC1, laminin subunit gamma 1; FBN1, Fibrillin 1; THBS1, Thrombospondin 1; FGFs, fibroblast growth factors; CD44, CD44 molecule; HGF, hepatocyte growth factor; LGALS9, Galectin 9; FGF2, fibroblast growth factor 2; STC1, Stanniocalcin 1; NFATC2, nuclear factor of activated T cells 2; BMP2, bone morphogenetic protein 2; PMAIP1, phorbol-12-myristate-13-acetate-induced protein 1; LMCD1, LIM and cysteine-rich domains 1; FGF7, fibroblast growth factor 7; IL15, interleukin 15; CRYAB, crystallin alpha B; KLRG2, killer cell lectin-like receptor G2; ACKR2, atypical chemokine receptor 2; CXCL12, C–X–C motif chemokine ligand 12; PDL1, programmed cell death ligand 1; PD1, programmed cell death 1; ADORA2B, adenosine A2B receptor.
