# Supplementary material for: Activation of Blood Vessel Development in Endometrial Stromal Cells In Vitro Cocultured with Human Peri-Implantation Embryos Revealed by Single-Cell RNA-Seq
Source: Life (Basel). 2021 Apr 21;11(5):367. doi: 10.3390/life11050367 (PMC8143346; doi:10.3390/life11050367)
Supplement: Supplementary file 1 [file life-11-00367-s001.zip › life-1155337-supplementary.pdf]

Supplementary materials:

Activation of Blood Vessel Development in Endometrial Stromal Cells In Vitro Cocultured with Human Peri-Implantation Embryos Revealed by Single-Cell RNA-Seq

Bo Lv †, Xiaoyu Xu †, Xunyi Zhang †, Lingbin Qi, Wen He, Lu Wang, Xian Chen, Luying Peng, Jinfeng Xue *, Yazhong Ji * and Zhigang Xue *

Table S1. Summary of number of individual EmSCs and DEGs between PRE and POST group.

EmSCs & Gene Number	Pre	Post	Total
**Cell Number**	24	57	81
**DEG * (Log2FC>1)**	569	1783	2352

* The number of DEG in this table represented the number of upregulated genes (FDR <0.05) in the individual groups.

**Table S2. The list of gene sets used in Figure 3E ("blood vessel development") and Figure S2 ("female pregnancy" and "angiogenesis").Pathways**	**Genes**
blood vessel development	*AAMP, ACVR2B, ANPEP, ATP2B4, BMP4, COL1A1, COL1A2, COL3A1, COL15A1, EPAS1, FAP, GATA6, NR4A1, HPGD, IL1A, CXCL8, LOX, LRP1, LTBP1, MMP14, NFATC4, ENPP2, PGF, PLCG1, PRKD1, PTGIS, PTGS2, SFRP1, TGFBI, THBS2, TNFAIP3, WNT11, RECK, ALDH1A2, HGS, CUL7, RAMP1, HEY1, ADGRA2, DLL1, EPN1, SUFU, SPHK2, TBX20, RHOJ, E2F8, COL18A1, RSPO3, CREB3L1, HSPB6, UNC5B, THSD7A, MIR199A2, MIR503*
female pregnancy	*ADRA2C, AR, BCL2, ESR1, GJA1, HPGD, HSD11B2, LIF, MMP9, OVGP1, PGF, PPARD, PTGIS, PTGS2, RECK, ENDOU, SLC38A3, EPN1, SPHK2, TLE6*
angiogenesis	*AAMP, ANPEP, ATP2B4, BMP4, COL15A1, EPAS1, FAP, GATA6, NR4A1, IL1A, CXCL8, MMP14, NFATC4, ENPP2, PGF, PLCG1, PRKD1, PTGIS, PTGS2, SFRP1, TGFBI, THBS2, TNFAIP3, RECK, HGS, RAMP1, HEY1, ADGRA2, DLL1, EPN1, TBX20, RHOJ, E2F8, COL18A1, RSPO3, CREB3L1, HSPB6, UNC5B, THSD7A, MIR199A2, MIR503*

Table S3. The list of primers used in the study.

Genes	Primer sequence (5′ to 3′)
RAMP1	AGAGGTGGACAGGTTCTTC<br>CTACACAATGCCCTCAGTG
LTBP1	CCCTAATGGTGAGTGTTTGA<br>ATTCCACCAGGACAGATTTC
LRP1	TTCACAAAGGAGACTACAGC<br>TCGATCTGATCCAGGTTACT

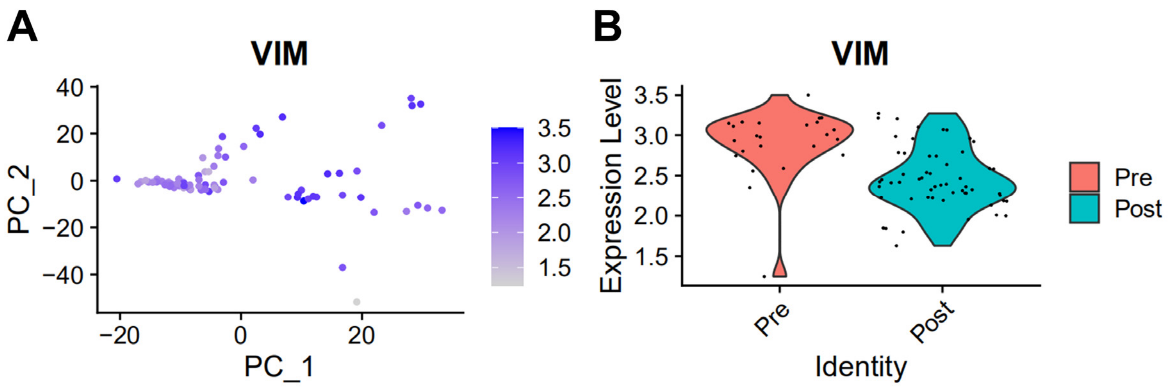

Figure S1. Characterization of endometrial stromal cells. Plot of vimentin (VIM) expression among all cells (A) and between the 'PRE' EmSCs and the 'POST' EmSCs (B).

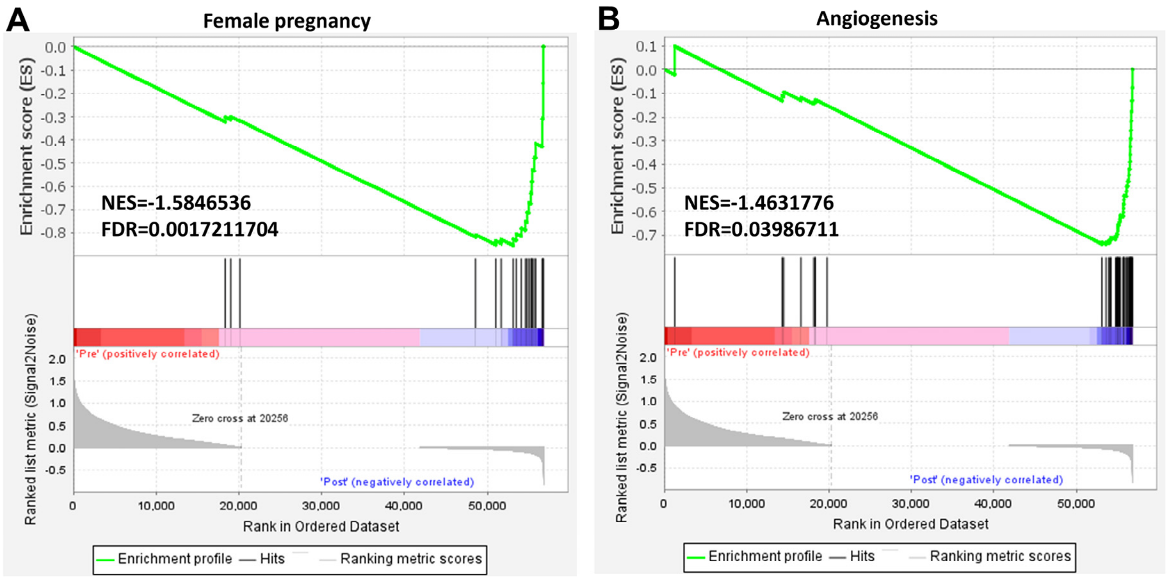

Figure S2. GSEA for female pregnancy gene set (A) and angiogenesis gene set (B).

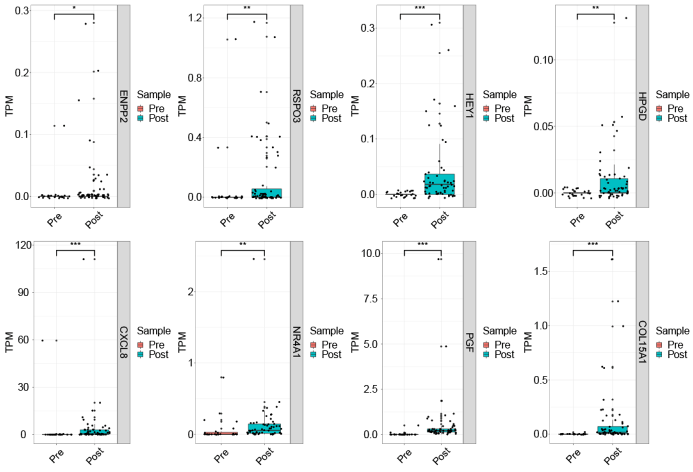

Figure S3. Boxplots showing other high expressed blood vessel development genes in POST EmSCs compared to PRE EmSCs using transcript per million (TPM). The asterisk means statistical significance (*$p < 0.05$, ** $p < 0.01$, *** $p < 0.001$). Dots represented the value of individual cell in each group. Genes (from left to right, top to bottom): ENPP2, RSPO3, HEY1, HPGD, CXCL8, NR4A1, PGF, and COL15A1.

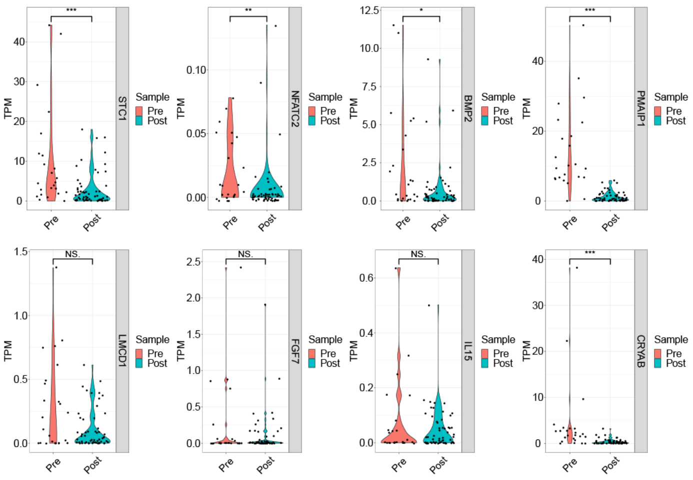

Figure S4. Violin plots showing endometrial phases related genes between PRE cells and POST cells using transcript per million (TPM). The asterisk means statistical significance (*$p < 0.05$, ** $p < 0.01$, *** $p < 0.001$); NS indicated no significance between two groups. Dots represented the value of individual cell in each group. Proliferative stromal genes (upper panel, from left to right): STC1, NFATC2, BMP2, and PMAIP1. Secretory stromal genes (below panel, from left to right): LMCD1, FGF7, IL15, and CRYAB.
